# Beyond heroism: a qualitative study on the lived experiences of nurses caring for patients with COVID-19 in Pakistan

**DOI:** 10.1186/s12912-023-01279-9

**Published:** 2023-04-06

**Authors:** Mahreen Afzal, Muhammad Abo ul Hassan Rashid, Florian Fischer

**Affiliations:** 1 Shaheed Zulfikar Ali Bhutto Institute of Science and Technology, Islamabad, Pakistan; 2 Institute of Public Health, Charité – Universitätsmedizin Berlin, Berlin, Germany; 3 Bavarian Research Center for Digital Health and Social Care, Kempten University of Applied Sciences, Kempten, Germany

**Keywords:** Pandemic, Pakistan, Nurses, Experiences, Survey, Thematic analysis, Corona, SARS-CoV-2

## Abstract

**Background:**

Healthcare professionals around the globe suffered severely during the COVID-19 pandemic. The present study aims to explore the lived experiences of nurses caring for COVID-19 patients in Pakistan.

**Methods:**

The study is a qualitative exploration of the lived experiences caring for patients with COVID-19 in Pakistan. This research was conducted in two government hospitals there. Face-to-face in-depth interviews were conducted amongst 30 nurses who had been selected using purposive sampling technique. Thematic analysis was applied to extract the themes from respondents’ answers.

**Results:**

By using thematic analysis, social response, impacts on physical and mental health, and experience of handling COVID-19 patients were extracted as major themes.

**Conclusions:**

The findings of this research are of immense importance in showing the impact of COVID-19 on mental and physical health, along with the social and personal consequences for nurses providing care to COVID-19 patients.

## Background

Healthcare professionals around the globe suffered severely during the COVID-19 pandemic [1, 2]. They not only suffered from mental effects such as anxiety [3, 4], depression [5], anger [6] and exhaustion [7], but also faced social and economic problems [8, 9]. Nurses make up a significant part of healthcare delivery, not least because of their devotion to work and the amount of time they spend providing care to COVID-19 patients [10]. Nurses have played a tremendous role not only in caring for COVID-19 patients, but also in curtailing the pandemic [11, 12]. Healthcare providers, especially nurses, who are working on the frontline have valuable experiences of handling and caring for COVID-19 patients [13–15]. In Pakistan, it has long been the general norm for the majority of the nurses to be females [16, 17]; and nursing is a common career choice for women [18, 19]. Most importantly, female nurses played a very significant part in effectively managing COVID-19. To gain an insight and in-depth information regarding the experiences of nurses working with COVID-19 patients, the present study aimed to complete an exploratory analysis of nurses’ experiences while managing the chronic situation of the COVID-19 pandemic. During the pandemic, as front-line fighters, the most vulnerable individuals were nurses [20]. It is worth mentioning that healthcare workers who care for patients with coronavirus are more likely to suffer from psychological problems including post-traumatic stress disorder [21], anxiety [22], depression [23, 24], and sleeplessness [25].

### Significance of the study

The main reason for conducting this research was to explore the kinds of problems and issues with which nurses providing care to COVID-19 patients were confronted. Lived experiences are basically explored and understood through qualitative research. Lived experiences are, in fact, a representation and understanding of a researcher related to human choices, experiences and options, and provide insights into how all these factors impact an individual’s perception of knowledge [26]. According to Honey et al. [27], study designs investigating lived experiences try to answer questions as to “How?”, “What?”, and “Why?”. Therefore, the meaning or theory can be extracted from the context in which people experienced the phenomenon [28]. This study was specifically designed to inquire about the experiences made by nurses providing care to COVID-19 patients at two government hospitals in Pakistan.

## Methods

### Procedures of the study

We adopted a purposive sampling technique while conducting this study. Participants were selected in terms of the inclusion criteria, and information was collected from the female nurses working in specially allocated treatment and isolation centres for COVID-19 patients and cared for them.

This study was conducted at the government hospitals in Islamabad (Polyclinic Hospital) and Rawalpindi (Benazir Bhutto Hospital). Both cities were among the hardest-hit districts in the second and third wave of the COVID-19 pandemic in Pakistan. Both cities take in people from all over the Pakistan, consisting of rural and urban populations. The population of this study was those nurses who provided care to the patients of COVID-19 at the government hospitals in the districts of Rawalpindi and Islamabad, Pakistan.

The sample size was not fixed a priori; instead, sampling could be continued until the data saturation point was reached. We managed to reach data saturation when 30 participants were being interviewed. Prior to the interview, approval to conduct the research was sought from the hospitals’ authorities together with the participants’ informed consent. Participants were informed about the reason for and significance of the investigation, and their written consent for participation was taken.

### Data collection

Qualitative data was collected with the help of an interview guide including research questions such as: What were your overall experiences in caring for patients with COVID-19? Could you describe how you deal with your life as a nurse taking care of patients with COVID-19 every day? How do people react towards you when they know you are a nurse in the hospital? How did you manage your personal/family life while performing your tough duties? What were the impacts of this toughest routine on your mental and physical health? How did you manage these problems? Individual face-to-face interviews were conducted in order to acquire the desired data from the participants.

### Data analysis

The procedure used to analyse data was thematic analysis, which is one of the most frequently used in the domain of qualitative research. Thematic analysis is performed through coding the acquired dataset in six different steps in order to create sub-themes and themes. All six phases are important and occur in sequence; each phase builds on the previous one [29]. These six phases applied in this study are the following:

1. *Familiarisation with the data (transcription of data)*: The collected data was first transcribed into written form because it was collected through face-to-face interviews. Moreover, this phase involved reading and re-reading the entire transcribed data.
2. *Generating initial codes*: On the basis of all the important features of the data, we generated codes by keeping in view their relevance to the research question. Coding was applied to the entire dataset. All relevant codes were kept for further stages of data analysis by extracting the unnecessary ones.
3. *Generating themes*: This phase was highly important because it involved investigating the extracted codes. Major, significant and broader sub-themes and themes were generated on the basis of these codes.
4. *Reviewing themes*: Themes were reviewed to ensure authenticity and proper utilisation according to the data. We also checked whether the themes answered the research question and in how far they were relevant to the generated codes. Moreover, proposed theories were reviewed in accordance with their support of the refined themes, which led to few changes in the generated themes.
5. *Defining and naming themes*: Themes were defined and named accordingly, keeping the scope and focus of each theme in consideration.
6. *Producing the final report*: After defining and reviewing the themes, we wove together the analytical narrative. The final report was written after conducting the analysis of the entire findings in relation to the existing literature.

### Ethical approval

The study was granted ethical approval by the Institutional Ethical Review Board of Shaheed Zulfikar Ali Bhutto Institute of Science and Technology, Islamabad, Pakistan (ref. no. IERB(7)/SZABIST-ISL(SS)/1,890,102/200,175).

## Results

The characteristics of the respondents are presented in Table 1. Almost all respondents in the sample belong to the age group 25–35 years. The minimum age was 25 years and the maximum 55 years. The nurses’ experience in providing healthcare services ranged from one to eight years, with one respondent having even 30 years of experience. The majority of nurses were married and living in nuclear families. All of them were female and performed their duties for COVID-19 patients for a period of one to four months. Nursing is a very common and general career for females in Pakistan [30, 31].

**Table 1:** Participants’ characteristics

ID	Age (in years)	Marital status	Designation	Family type	Shift	Work experience (in years)	COVID-19 duty duration (in months)
P1	30	Married	Nurse	Nuclear	Morning	4	2
P2	27	Unmarried	Nurse	Nuclear	Morning	2	3
P3	30	Married	Nurse	Nuclear	Morning	5	2
P4	26	Unmarried	Nurse	Nuclear	Morning	1	1
P5	30	Married	Nurse	Nuclear	Morning	5	4
P6	28	Married	Nurse	Nuclear	Morning	3	3
P7	26	Unmarried	Nurse	Nuclear	Evening	1	1
P8	27	Unmarried	Nurse	Nuclear	Morning	2	2
P9	28	Married	Nurse	Nuclear	Morning	3	2
P10	28	Unmarried	Nurse	Joint	Morning	3	3
P11	27	Unmarried	Nurse	Joint	Evening	2	2
P12	27	Unmarried	Nurse	Nuclear	Morning	2	2
P13	55	Married	Head nurse	Joint	Morning	30	1
P14	30	Married	Nurse	Nuclear	Morning	5	4
P15	26	Married	Nurse	Joint	Morning	1	1
P16	34	Married	Nurse	Joint	Morning	7	1
P17	30	Married	Nurse	Joint	Morning	5	5
P18	25	Unmarried	Nurse	Nuclear	Evening	1	1
P19	26	Unmarried	Nurse	Nuclear	Morning	1	1
P20	28	Unmarried	Nurse	Nuclear	Evening	2	2
P21	28	Unmarried	Nurse	Nuclear	Evening	2	2
P22	32	Married	Nurse	Nuclear	Morning	6	1
P23	32	Married	Nurse	Nuclear	Morning	6	2
P24	30	Married	Nurse	Nuclear	Morning	5	2
P25	30	Married	Nurse	Nuclear	Morning	5	1
P26	30	Married	Nurse	Nuclear	Evening	5	1
P27	36	Married	Nurse	Nuclear	Morning	8	2
P28	35	Married	Nurse	Nuclear	Morning	8	2
P29	32	Married	Nurse	Nuclear	Morning	6	2
P30	30	Married	Nurse	Nuclear	Evening	5	1

The themes and sub-themes extracted while applying thematic analysis are presented in Table 2 and described as follows.

**Table 2:** Themes and sub-themes for data analysis

Themes	Sub-themes	Codes
Social response	Strong support system Strong social bonding	Family support, appreciation by the society, encouraging attitude, supportive spouse, cooperative colleagues, motivation
Impact on mental health	Fear Anxiety Depression	In the beginning, anxiety, depressive thoughts, fear of the disease, stress of carrying germs of the disease (family concern)
Impact on physical health	COVID-19 positive Fatigue Breathing issues	Fatigue, weakness, increased workload, sleeplessness, fever, difficulty in breathing, lack of appetite
Experience of handling contagious diseases	Professional requirements Serving the humanity	Professional requirement, sympathetic attitude, honest to duty, sense of serving the humanity, proud to be a nurse

### Social response

The most important component of this study was to inquire amongst the interviewees about the responses of society, friends and family towards them during this pandemic. Healthcare workers suffered the most from the COVID-19 pandemic because they sacrificed their personal lives and responsibilities, own health and preferences to serve the masses and to be available for handling patients with COVID-19 during this pandemic. The statements of the nurses who participated in this research regarding social response and attitudes towards them are discussed in two further sub-themes, which relate to a strong support system and strong social bonding.

#### Strong support system

This study aimed to explore how people reacted towards nurses in the knowledge that they were constantly exposed to a contagious disease. The majority had positive experiences regarding the societal response towards them, despite the different impacts of the pandemic on individuals:

“I tested positive with COVID-19, home quarantined for one month; during all this my family supported me a lot. Now I am performing my duty with the full-time support and assistance of my family. My family is side by side with me. They did not leave me alone in this battle to fight with. We still cannot overcome the crisis and win this battle without constant support of our families.” (P13).

“I was pregnant last year and tested positive with COVID-19. Although it could be unmanageable for me to handle the situation, but, I must say, my husband, in-laws and all my family supported me a lot. So, I managed everything well and fulfilled all my personal responsibilities with the support of my husband. Even at work, my fellow nurses and all other staff helped me to manage my things while I was on duty in COVID-19 units.” (P16).

The responses of the participant nurses clearly indicate that they received full support from family members when they were performing tough duties in the hospital. Nurses mentioned that it was not an easy thing to manage their responsibilities during the worst crisis of their career. Not only did their family members encourage them, but their colleagues also proved to be a great source of assistance and support in handling the unforeseen situations.

#### Strong social bonding

Although the situation might differ compared to other countries or even to nurses from other hospitals, we ascertained from the experiences of the study participants that they had a robust support system and strong social bonding. The spouses of married nurses supported them in managing their duties and this happened because of their strong bonding. However, there were some people who avoided interacting with them, but it was all because of the fear of contracting the virus. Respondents in the study were also concerned about the health, protection and safety of their family members. Therefore, they tried not to visit them for an extended period of time:

“I myself avoided frequent social interaction in order to keep people safe if I carry the virus. My husband helped me a lot to remain honest with my profession. On both sides I had my own people to care for.” (P14).

Participants mentioned that they received support from the hospital to help limit social interactions:

“I received full cooperation while I was serving in the COVID-19 intensive care unit. I stayed in a nursing hostel to limit my interaction with people, just to keep them safe. I avoided visiting my family for a long time. […] When I am on duty in corona units, I prefer to stay in a nursing hostel because I assume it is better for my family.” (P21).

### Impact on mental health

Any stressful situation impacts on mental health and well-being. This has been particularly visible during the COVID-19 pandemic. All participants of this study took maximum safety measures while interacting with their families and other people around them. There was little known about the disease/virus in the beginning so that ambiguity caused initial upset and stress among them; but later on, they managed to cope with the situation. However, the early situation impacted their mental health and they faced and had to manage some psychological problems such as fear, anxiety and depression.

#### Fear

Nurses were fearful of COVID-19, particularly in the initial stages of the pandemic. Mostly, nurses were more concerned about their families than themselves, because they tried to avoid contracting the disease and transferring it to their children and other family members:

“If I say that I wasn’t fearful of the disease, I must be lying to you. Yes, I had fears.” (P17).

“Being an active participant of the medical field and exposed to COVID-19 patients, I didn’t want to become a source of transmitting the disease to my family and other people around me.” (P26).

Fear was common among many respondents at the very beginning of the pandemic.

“Initially, I was concerned about my performance and I literally wanted to overcome my fear because I didn’t want to compromise on my duties.” (P28).

“Sometime when I had symptoms of normal fever, I thought what happen if I test positive for this disease? Who would take care of my kids even if I die?” (P27).

The responses of the nurses clearly indicate that they experienced fear while performing their duties in the initial stages of the pandemic. These fears might have badly impacted their mental health. Nurses had gone through such experiences at the very beginning of the outbreak of COVID-19, when there was uncertainty and ambiguity. Later on, they not only controlled their own fear but also helped and guided others to behave properly.

#### Depression and anxiety

The nurses’ experiences reveal that they were aware of the impacts of their mental condition on their job and the quality of care that they provided to the patients. Furthermore, they were fully aware and understood the severity of these conditions while they were handling COVID-19 patients.

Respondents described various factors behind their anxiety and stressful conditions, including restless routine, haziness about the treatment and nature of the disease (during initial stages), severity of the disease, stress for the event of grievous circumstances, and overall, a restless and questioning environment. There was an initial feeling of sudden emergency and consecutive stress of an unfortunate environment. The majority of nurses narrated that they faced anxiety because in the initial stages of the outbreak there was no clarity of the existence and treatment of the disease even among healthcare providers:

“We didn’t know much about the disease. How could we stay calm while witnessing people dying with coronavirus when the cases were on their peak in the country… Even the attendants of patients were unable to bid them last farewell. It was such a heart wrenching and emotional thing to see and pay focus on performing our duties.” (P18).

“I won’t deny the fact that I was panicked… I used to get panic… I still remember… Although we see people dying every day, the situation in the case of the coronavirus was entirely different. People should not treat it as a joke.” (P4).

It was highly difficult for the nurses to see people dying without having their loved ones with them in their last moment. Witnessing these worst situations made nurses emotional and more empathetic towards their COVID-19 patients:

“We are used to handle situations of emergency. However, this pandemic made us experience many worst things. We see people dying of it and their loved ones cannot be with them! It is difficult to handle such emotional situations sometimes.” (P21).

COVID-19 changed the lifestyle of many people. When nurses were asked to explain the situation of the pandemic, they frequently managed the psychological impact and their experiences related to it. Nurses, more specifically married ones, were quite worried about their children. They did not want to bring them into an undesirable situation:

“My family was my biggest concern and I did not want to become the cause of their pain if they might get the disease. Even now, every time when I go home, I don’t interact with my family unless I change my uniform, take a proper bath and proper sanitization.” (P20).

“Being a mother, I had so much to do for my kids and I thought what will happen if I die soon. These thoughts were enough to put me under depression. I have little kids to look after. I cannot rely on anyone in this matter. This was my biggest worry while handling patients in COVID-19 special units.” (P30).

The experiences of nurses handling patients with a contagious disease have highlighted the impacts of this ongoing pandemic on their lives, responsibilities and mental wellbeing.

### Impact on physical health

Nurses are frequently called “superheroes” or “life savers” by the public as a result of the tasks that they successfully fulfilled during the COVID-19 pandemic. Appreciation by the people added much worth and appeasement in the healthcare professions. However, at the same time, it put more pressure on healthcare workers, by leaving no chance of mistake from their end. Furthermore, fulfilling the tasks puts pressure on them and negatively impacts upon their physical health.

#### COVID-19 positive

In the case of Pakistan, many healthcare workers sacrificed their lives while handling COVID-19 patients. All respondents of this study performed their duties in specially allocated COVID-19 units. A few nurses even became COVID-19 positive themselves. The responses of the nurses related to contracting the disease are described below:

“I tested positive with coronavirus last year and home quarantined myself for 15 days. I not only recovered from it but I am absolutely fine. Although I belong to the upper age group, still I don’t have major complications after contracting the virus – except for a few ones.” (P13).

“I tested positive last year while I was pregnant and I was really upset about how to manage this toughest routine while expecting a baby.” (P16).

Furthermore, the study participants also mentioned that a few of their staff members sacrificed their lives in the line of duty.

#### Fatigue

When nurses were asked about the impact of COVID-19 on their physical health, almost all of them spoke about it and narrated their related experiences. The pandemic has altered the life of every individual. Due to the heavy workload and excessive precautionary measures to handle the patients along with standard operating procedures (SOPs), the lives of nurses also became hectic: They had to wear protective gear (such as face masks, gloves, protective gowns), sanitise everything, and change their clothes every day as soon as they reached their hostels or homes. Initially, nurses had to perform excessive work that became a cause of sleeplessness. Heavy workload in hospitals and tough routines following SOPs may lead to extreme fatigue. All nurses – particularly those who were infected with COVID-19 themselves – experienced weakness:

“I felt extreme weakness and fatigue while I was performing my duties in COVID-19 wards.” (P30).

“I did not focus on my diet while I was serving in a COVID-19 unit. That was also the reason behind my weakness. Actually, because of extensive sanitisation and safety protocols, I got so [much] exhausted and had no desire to do anything but relax.” (P29).

Furthermore, study participants mentioned that due to the work burden they could not focus on their own health and had fatigue. Carrying personal protective equipment increased their work and made them exhausted:

“Although I was not fearful, but yes… I had fatigue while I was serving in COVID-19 units because of following SOPs and excessive precautionary measures, which is even today mandatory to follow by every nurse who handles patients with COVID-19 in the hospital. Moreover, I do not take proper care of myself, but now I realised that self-care is highly important in order to handle this situation. Otherwise, I would be of no use. And I don’t want that to happen.” (P2).

In fact, the respondent nurses mentioned that excessive protective measures and adopting COVID-19 SOPs increased their work. Physical fatigue was normal in emergencies, but the prolonged duration of COVID-19 actually brought negative impacts on the health of nurses:

“Body fatigue is a normal thing for us in situations of emergencies and workload. But this time it was a mental fatigue as well. Initial uncertainty, misinformation and myths played a huge role in increasing our duty, because it took us a lot of energy and time to educate people that their lack of care could further spread the disease. Sometimes, even now it becomes really difficult to handle attendants of the patients in terms of following safety protocols and implementing social distancing.” (P10).

### Experience of handling contagious diseases

When nurses were asked about their experience of handling patients with a contagious disease such as COVID-19, two themes emerged from data which relate to professional requirements and (nurses’) intensions to serve humanity.

#### Professional requirements

All study respondents had experiences of handling contagious diseases, caused by e.g. the Human Immunodeficiency Virus (HIV), Hepatitis (A, B and C), dengue and then most importantly COVID-19:

“We became used to this situation because it is our profession. For me, my profession is more than anything else. I have no regrets joining this. At least I am satisfied with what I am doing.” (P4).

The participants in this study highlighted the fact that they encountered initial fear because of the uncertainty and misconceptions about the virus. But, later on, they became familiar with the situation and took it as their professional responsibility:

“We joined this profession to serve people. I even handled patients who were suffering from AIDS and cancer in my entire career. I have seen the pain and misery of people. However, this time it was not only for us, but the entire world was battling hard with it. Even though few countries have got some hold on it. But we can clearly see the all-time worst situation of coronavirus in India. It is a requirement of our profession and we are always here to serve in any situation.” (P13).

“Even having suffered from this COVID-19 pandemic by myself, I am still motivated and good to perform my duty without any fear.” (P13).

Nurses showed that they were motivated to cope with the crisis because it was their duty to do so:

“Yes, when there is some uncertainty about the disease, like this pandemic, in the very beginning we had some fearful thoughts, but we know that it is our profession and we have to do our job no matter what happens.” (P25).

The nurses were motivated to manage their work and family life along with battling COVID-19 on the front line.

#### Serving humanity

Nurses join this profession knowing its hardness, and in case of pandemics and emergencies they know that they have to work wholeheartedly and provide their serves to the public. Almost all nurses responded that no matter how fearful and restless they get in the beginning of any emergency, they have to work to manage themselves and overcome their fear to serve the people in need of care:

“We took an oath to serve humanity, so we know it is the requirement of our profession, and we came into this profession knowing the severity and demands of it. Neither can we run away from this situation nor should we!” (P24).

The nurses participating in this study were all ready to serve humanity. They were passionate to work during the pandemic and motivated enough to beat it:

“I was not at all fearful. Not even in the beginning of the pandemic in Pakistan. I worked in COVID-19 special units. My only concern was the pain and misery of the people who were dying of it. I was very sympathetic towards them. I do my best to serve my people. Even if I have to devote my entire life to serve humanity, I would literally love to do that! It might be surprising for you, but I am like this. I feel the pain of my patients.” (P2).

Irrespective of initial fear and anxiety, nurses were more concerned about their professional performance and providing their best services to the patient than anything else.

## Discussion

The present study has highlighted the experiences of nurses who cared for patients with COVID-19 in Pakistan. Nurses served wholeheartedly and with full devotion throughout the pandemic while standing on the front line in terms of patient care, providing COVID-19 service, and managing the crisis. Nurses stood firmly against the pandemic and sacrificed their lives in the line of duty. They faced an extreme workload due to the pandemic and experienced severe anxiety, mental and physical fatigue.

The study revealed that in the face of psychosocial pressure, fear of death, anxiety and depression, the nurses received strong social support from their families, friends and community members. The participants of this study personified a strong gesture of social bonding, which they experienced because of the heroic duties that they performed during the pandemic.

The findings of this research clearly show that nurses went through fear and anxiety at the beginning of the pandemic. In addition to this, the persistent exposure of healthcare workers to stressful situations such as witnessing the suffering and pain of patients who were dying with the disease amplified anxiety and fear [24].

It is evident in many studies that COVID-19 significantly affected the psychological and mental health of the general population, but more especially nurses [32]. Frontline health providers, particularly nurses, were more vulnerable to deterioration in their mental health [33, 34]. This is consistent with previous research, which explored the fear among health care providers [35, 36]. As previously observed by Sakib et al. [37], that the pandemic significantly created fear and depression among healthcare workers, this study revealed that participants experienced the same (i.e. the fear of being quarantined, losing family and friends, and, more importantly, of being socially rejected or stigmatised), but at the same time also a strong social bonding and overwhelming social support from their family and community members.

Among all healthcare providers, nurses had the greatest exposure to and closest contact with patients. According to statements by study participants, they were not much concerned about their own lives, but rather their families were their main concern. Another main finding was that nurses did not want to compromise on the care they provided to their patients. This leads to adverse impacts upon physical and mental health [38]. Therefore, nurses need resilience to tackle the additional responsibilities and challenges presented by the COVID-19 pandemic [39]. Nurses showed extreme resilience and professionalism to tackle these difficulties.

Globally, it has been witnessed that healthcare professionals sacrificed their lives while fighting on the front line against COVID-19 [40, 41]. One of the most important findings of this study was the willingness of nurses to serve humanity. Although they suffered from stress and fear during the initial stages of the disease, they adapted themselves to the situation later on. Nurses experienced a sense of accomplishment and felt better as soon as they accepted the situation. Despite all that difficulty and uncertainty, they were concerned about the health of their patients. This led to the attribution of being a “superhero” [42].

### Strengths and limitations of the study

This research focused solely on nurses who were providing care for patients with COVID-19, and the study area was limited to one hospital each in Islamabad and Rawalpindi. It was extremely difficult to conduct the interviews because of the surge in cases of COVID-19 in Pakistan, more specifically during its third wave. The situation of the lockdown also raised a barrier to and placed limitations on initiating the data collection process. It took a lot of time and effort just to conduct the interviews and collect data because the possibility of interaction with nurses was limited due to their workload and safety issues. Considering the qualitative research approach, it was also a challenge for our team to analyse the data and present a logical conclusion due to the restricted generalisation of the findings in comparison to quantitative research. The major fears of the medical practitioners that have been highlighted by this research include the risk of being on the front line during this pandemic and having to stay socially isolated from their families to keep them safe. Also, many of the participants mentioned that their fears increased because of the uncertain nature of this pandemic.

## Conclusions

Globally, the COVID-19 pandemic placed entire healthcare systems under extreme pressure. Healthcare professionals were more prone to COVID-19 because they served on the front line against this devastating pandemic and sacrificed their lives as well as developing several mental, physical and psychological issues. Social responses, the impact of COVID-19 upon mental as well as physical health, and the experiences of nurses handling contagious diseases were the main themes of this research. During the COVID-19 pandemic, nurses performed tough duties. Intense working environment and workload can be a source of insomnia for healthcare professionals, especially nurses. Thus, the management of the hospitals concerned should sort out such situations in a timely manner in order to prevent the core segment of the medical field from becoming a prey to insomnia and professional stress. The safety and well-being of nurses should be given priority. The well-being of healthcare providers should be government’s most important priority because their well-being ensures their best performance at work. Furthermore, an increase in workload takes away time and energy from family, particularly small children and senior dependants such as aged parents. Fears of exposing families and children to diseases after returning from work are similarly linked to higher anxiety among nurses. Future researchers should adopt broader qualitative research methodology. For instance, ethnomethodology, ethnography and phenomenology. It is suggested that future researchers should consider working on and exploring the nurses’ core competencies in order to find out how well they were prepared to handle emergencies such as COVID-19 and similar situations.

## Data Availability

In this qualitative study, the raw data cannot be made publicly available for data safety reasons. However, data is available from corresponding author upon reasonable request.

## Abbreviations

COVID-19: Coronavirus disease 2019
SOP: Standard operating procedure
